# Editorial: Human reactions to artificial intelligence with anthropomorphic features

**DOI:** 10.3389/fpsyg.2026.1873310

**Published:** 2026-06-30

**Authors:** Sara Ventura, Maurizio Mauri, Janie Brisson

**Affiliations:** 1 Instituto Polibienestar, University of Valencia, Valencia, Spain; 2 Department of Medical and Surgical Science, University of Bologna, Bologna, Italy; 3 Research Center in Communication Psychology (PSICOM), Catholic University of Sacred Heart, Milan, Italy; 4 Département d'éducation et pédagogie, Université du Québec à Montréal, Montreal, QC, Canada

**Keywords:** AI anthropomorphism, artificial intelligence (AI), embodiment, human-robot interaction, social cognition, users perception

The rapid proliferation of artificial intelligence (AI) technologies has fundamentally transformed how humans perceive, interpret, and interact with social information (Kuzior and Kwilinski, 2022). The present Research Topic brings together 12 contributions that collectively examine how AI-generated agents, ranging from avatars and virtual influencers to chatbots and robots, reshape socio-cognitive processes, emotional engagement, and human–machine relationships. The overarching aim of this Research Topic is to bridge traditional theories of social cognition with emerging AI-driven environments, while critically assessing both the opportunities and challenges that arise when artificial agents become embedded in everyday social contexts. A central objective across the contributions is to understand whether and how artificial agents can replicate, augment, or distort core human social processes. Several articles converge on the premise that social cognition is inherently multimodal and context-dependent, requiring the integration of cues such as facial expressions, language, and social categories. In this regard, the use of AI-generated stimuli represents a methodological turning point. For instance, experimental work employing AI-generated videos demonstrates that dynamically integrated cues can elicit nuanced judgments of plausibility and emotional intensity, highlighting the sensitivity of human observers to congruence across modalities (Kreifelts et al., 2007). This shift from static to dynamic stimuli enhances ecological validity while enabling unprecedented experimental control, thereby opening new avenues for investigating social information processing. Beyond methodological innovation, the Topic also addresses the experiential and relational dimensions of human–AI interaction. A recurring theme concerns the extent to which artificial agents are perceived as social actors. Drawing on longstanding frameworks such as the Media Equation, several contributions show that individuals readily attribute intentions, emotions, and mental states to AI systems, particularly when these systems exhibit human-like features or behaviors (Coghlan, 2024). However, this attribution is neither uniform nor unproblematic. Reviews and theoretical papers emphasize that while artificial agents can approximate aspects of human interaction, important differences remain, necessitating careful consideration of experimental design, measurement specificity, and interpretative boundaries.

The role of emotional expression emerges as another key focus. Contributions examining human-like virtual influencers reveal that emotional cues are central to fostering user engagement, primarily through the mediation of empathy. Specifically, perceptions such as authenticity, presence, attractiveness, and escapism shape both cognitive and affective empathy, which in turn drive behavioral engagement. These findings underscore that effective human–AI interaction is not solely a matter of technological sophistication but also of psychological resonance. Emotional expression, when perceived as authentic, can strengthen relational bonds even in explicitly artificial contexts (Nass et al., 1994).

At the same time, several articles critically interrogate the notion of “artificial empathy.” Empirical investigations into AI-generated outputs highlight potential discrepancies between perceived and genuine empathy, raising questions about authenticity, moral evaluation, and user trust. This line of work contributes to an emerging discourse on the “illusion of empathy,” suggesting that while AI systems can simulate empathic responses, users may differentiate between surface-level expression and deeper moral or emotional understanding. Such findings have significant implications for domains where empathy is pivotal, including healthcare, education, and customer interaction (Glikson and Woolley, 2020).

Design considerations further complicate the landscape of human–AI interaction. The phenomenon of the uncanny valley is repeatedly identified as a critical boundary condition. Highly human-like agents can enhance social presence and engagement, yet they may also evoke discomfort or skepticism when perceived as overly artificial or deceptive. Contributions in this Research Topic suggest that optimal design lies in balancing anthropomorphism with transparency, ensuring that artificial agents remain relatable without triggering unease. This insight reinforces the importance of interdisciplinary approaches that integrate psychological, technological, and ethical perspectives (Wu et al., 2024).

Collectively, the articles also highlight the importance of context in shaping human responses to AI. Social norms, cultural frameworks, and situational expectations influence how artificial agents are interpreted and accepted. For AI systems to function effectively as social partners, they must align with these contextual factors, facilitating interactions that are perceived as natural, trustworthy, and meaningful. This contextual sensitivity is particularly relevant as AI systems increasingly operate in domains traditionally reserved for human interaction.

Despite significant advances, the contributions in this Research Topic also underscore several unresolved challenges. Methodological limitations, such as small sample sizes, homogeneous participant pools, and platform-specific analyses, constrain the generalizability of findings. Moreover, conceptual ambiguities persist regarding the nature of empathy, the validity of social-cognitive measures, and the extent to which human–AI interactions can be meaningfully compared to human–human interactions. Addressing these challenges will require more robust experimental designs, cross-disciplinary collaboration, and the development of refined theoretical frameworks.

In a broader context, this Research Topic situates itself within a transformative moment for social cognition research. The integration of AI into social environments not only provides new tools for studying human behavior but also fundamentally alters the phenomena under investigation. Artificial agents are no longer mere stimuli; they are active participants in social ecosystems, capable of influencing cognition, emotion, and behavior in complex ways. This dual role, as both object and agent of study, necessitates a rethinking of traditional assumptions and methodologies.

In conclusion, the 12 contributions assembled here collectively advance our understanding of social cognition in the era of artificial intelligence. They demonstrate that while AI technologies offer powerful opportunities to model and investigate social processes, they also introduce new layers of complexity that challenge existing theories and practices. By framing these developments within a coherent research agenda, this Research Topic lays the groundwork for future inquiry into how humans and artificial agents can coexist, interact, and co-evolve within increasingly hybrid social worlds.
